# The Biosimilar Revolution: Assessing the European Union's Approach to Biosimilarity, Interchangeability, Patient Access, and Its Market Analysis

**DOI:** 10.7759/cureus.68103

**Published:** 2024-08-29

**Authors:** Gokul S, Sudheer Kumar, Kamaraj R

**Affiliations:** 1 Pharmaceutical Regulatory Affairs, SRM College of Pharmacy, SRM Institute of Science and Technology, Chennai, IND

**Keywords:** patient access, interchangeability, biosimilarity, eu regulatory framework, biosimilars

## Abstract

The biosimilar revolution alters the biopharmaceutical environment, delivering an appropriate strategy for improving accessibility and cutting healthcare costs. The European Union (EU) has established a broad regulatory structure to ensure the proper and efficient use of biosimilars. This review examines the EU's approach to biosimilarity, interchangeability, and patient access, encompassing the legal framework, scientific considerations, market dynamics, and patient viewpoints. The EU's regulatory system has developed to accommodate biosimilar development, approval, and adoption difficulties. Biosimilarity involves demonstrating comparability to the reference product, whereas interchangeability necessitates a more nuanced approach. Patient access is affected by pricing, reimbursement, and education. Employing a mixed-methods approach, this review combines an evaluation of regulatory documents, scientific literature, and market survey analysis. This review looks at the accomplishments and obstacles of the EU's strategy, identifying areas for advancement and chances for further development. This review intends to give significant insights to stakeholders, such as policymakers, producers, medical professionals, and patients, by examining the EU's biosimilar revolution. This critique helps to shape initiatives to improve the EU's approach, boost patient access, and promote sustainable healthcare systems. Ultimately, this review demonstrates that the EU's approach to biosimilars has successfully increased market growth and suggested suitable areas for development.

## Introduction and background

The biopharmaceutical industry is undergoing a significant transformation with the emergence of biosimilars, revolutionizing the way we approach healthcare. Biosimilars, similar versions of already-approved biological medicines, offer a promising solution to increase accessibility and reduce healthcare costs. European member states have been at the forefront of embracing this revolution, establishing a comprehensive regulatory framework to ensure the safe and effective adoption of biosimilars. As the European Union (EU) continues to navigate the complexities of biosimilar development, approval, and uptake, it is crucial to assess its approach to biosimilarity, interchangeability, and patient access. This review will provide suggestions for the upgrade of the current framework, identifying areas for improvement and opportunities for growth. The EU's regulatory framework for biosimilars, established in 2003, has undergone significant revisions, reflecting the evolving landscape of biotechnology and clinical practice. The European Medicines Agency (EMA) plays a vital role in ensuring the quality characteristics and safety assessment of biosimilars through rigorous approval processes [1].

Biosimilarity, a cornerstone of biosimilar development, requires demonstrating resemblance to the reference biologics in terms of critical product aspects. The EU's approach to biosimilarity has been shaped by scientific advancements, clinical experience, and stakeholder feedback.

Interchangeability, a critical aspect of biosimilar adoption, refers to the ability to change between the reference product and the manufacturer's product without compromising safety or efficacy. The EU's stance on interchangeability has evolved, acknowledging the need for a more nuanced approach [2].

Patient access, a key driver of the biosimilar revolution, is influenced by various factors, including pricing, reimbursement, and education. The EU has implemented measures to enhance patient access, but challenges persist.

Employing a mixed-methods approach, this analysis combines a systematic review of regulatory documents, scientific literature, and industry reports taken from journals and the official EU regulatory agency website listed in the reference.

Despite the EU's supportive regulatory framework, biosimilar adoption faces significant challenges and controversies. Stakeholder resistance, particularly from originator companies and some healthcare professionals, hinders market penetration. Concerns about biosimilarity, interchangeability, and safety fuel skepticism, leading to hesitancy in prescribing and using biosimilars.

Additionally, market barriers, such as patent litigation, aggressive pricing strategies, and limited education and training, impede biosimilar uptake. Patient organizations and payers may also have varying levels of awareness and acceptance, affecting demand. Moreover, the EU's diverse healthcare systems and reimbursement policies create uneven market access. Some member states have implemented measures to promote biosimilar use, while others lag behind. The lack of standardized guidelines and communication strategies further exacerbates the issue.

Addressing these challenges requires a multifaceted approach involving education, evidence generation, and policy interventions. Encouraging collaboration among stakeholders, improving transparency, and fostering a competitive market environment can help overcome resistance and increase biosimilar adoption, ultimately enhancing patient access to affordable, effective treatments.

This review will explore the EU's approach to biosimilarity, interchangeability, and patient access, exploring the regulatory framework, scientific considerations, market dynamics, and patient perspectives. By examining the successes and challenges, we can identify opportunities for improvement, ultimately shaping the future of biosimilars in the EU [1,3].

## Review

Regulated manufacturing of biosimilars

Manufacturing biosimilars is more challenging than developing chemically derived molecules. Biotechnology provides the basis for the production of the vast majority of biological medications, which often use multifaceted biological systems and recombinant DNA. EU law sets stringent regulations in the manufacturing of biosimilars: EU manufacturers must possess an authorization license and are bound by law to follow good manufacturing principles that rely upon protocols for producing a biosimilar of established quality [4].

Regulatory bodies in the member states execute periodic reviews of manufacturing facilities to ensure that they meet Good Manufacturing Practice (GMP) criteria. If any production stages occur beyond the concerned member state, other producers and market distributors must adhere to the stringent regulations and be subject to frequent inspections. Some GMP standards for biosimilars have been modified to account for their unique characteristics (e.g., the use of suitable aseptic procedures and storage) [2,5].

Approval of biosimilars in member states

**Figure 1:**
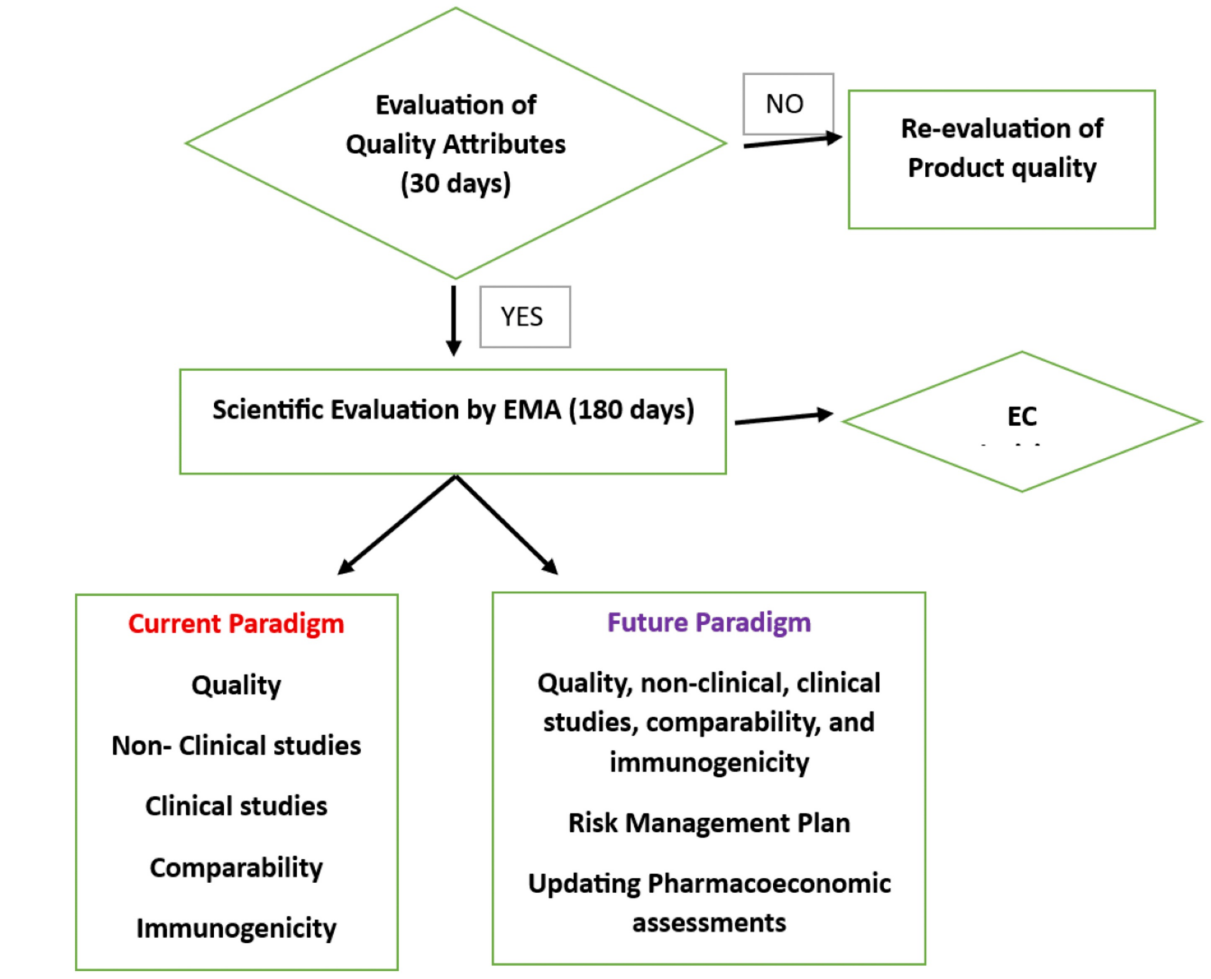
Approval of biosimilars in member states This image was created by Mr. Gokul S.

Biosimilars and products of particular indications, such as oncology and immune illnesses, need EU approval via EMA's "centralized approach." Because biosimilars are produced using biotechnology, almost all of those authorized for use within the EU were centrally approved. Some biosimilars, such as low-molecular-weight heparins produced from swine intestinal mucosa, may get national approval. When a business files for marketing permission at EMA, data are examined by "EMA's technical panels on human medicines and safety" (the CHMP and PRAC), as well as "EU experts on biological medicines (the Biologics Working Party) and biosimilar specialists (the Biosimilar Working Party)." EMA evaluation yields an unbiased concern, which is then forwarded to the "European Commission," which eventually provides an EU member state registration approval [6]. The flow diagram of the approval process is shown in Figure 1.

Data requirements for approval

Biosimilars are licensed when investigations on pharmaceutical quality, safety, and effectiveness show that the benefits exceed the dangers. A good benefit-risk balance for any biological therapy with a novel active component is evaluated primarily by proof of safety and effectiveness in pivotal human studies, which are backed by strong quality assurance data and preliminary data. A good benefit-risk balance for biosimilars is dependent on showing biosimilarity or the active substance's strong resemblance to the reference drug. This is accomplished by rigorous comparison tests with the reference drug, which are based on reliable pharmaceutical quality data. The biosimilar's strong resemblance to the reference drug allows it to rely on its effectiveness and safety experience [7].

The approval of biosimilars raises a complex array of ethical considerations, which must be carefully navigated to ensure the safety, efficacy, and accessibility of these medications. As biosimilars are designed to be highly similar to reference products, subtle differences may exist, and their impact on human subjects must be carefully evaluated. Informed consent is a critical ethical consideration in biosimilar trials. Participants must be fully aware of the potential risks and benefits of participating in these studies, including the possibility of receiving a biosimilar rather than the reference product. This requires transparent communication about the nature of biosimilars, their potential differences from reference products, and the uncertainties associated with their use.

Safety and efficacy are paramount ethical concerns in biosimilar approval. While biosimilars are designed to be highly similar to reference products, subtle differences may exist, and their impact on human subjects must be carefully evaluated. Regulatory agencies must ensure that biosimilars are safe and effective for human use and that any potential risks are mitigated through robust clinical trials and post-marketing surveillance.

Vulnerable populations, such as patients with rare diseases or limited treatment options, may be disproportionately affected by biosimilar approval. These individuals may have limited access to reference products or be more susceptible to potential differences between biosimilars and reference products. Regulatory agencies and manufacturers must prioritize the protection of these vulnerable populations, ensuring that biosimilars are safe and effective for their use.

Data transparency is another critical ethical consideration in biosimilar approval. Clinical trial data must be transparent, accessible, and accurately represent the safety and efficacy of biosimilars. This requires robust reporting of clinical trial results, including negative or inconclusive findings, to ensure that healthcare professionals and patients have access to accurate information.

Finally, the ethical considerations surrounding biosimilar approval must be balanced against the need for accessible and affordable medications. Biosimilars have the potential to increase access to life-saving medications, particularly in resource-constrained settings. Regulatory agencies and manufacturers must prioritize the development and approval of biosimilars that meet rigorous ethical standards while also promoting accessibility and affordability [2,7].

Approach to demonstrate biosimilarity

The establishment of biosimilarity between a biosimilar and a reference biologics includes an evaluation of the impact of any observable variations between the two components but not an independent determination of the proposed product's safety and efficacy. EMA suggests that manufacturers use a step-by-step approach to producing the scientific evidence and information required to promote an example of biosimilarity. At each phase, the sponsor should assess the level of remaining doubt regarding the biosimilarity of the component being considered and choose the next steps to resolve that ambiguity. Wherever feasible, research should be planned to make the most contribution to showing biosimilarity. A clinical immunogenicity study might give further information regarding the proposed product's safety profile [8,9].

The step-by-step strategy should start with an operational and structural assessment of intended and reference biologics, which may act as the base for a biosimilar research program. The comparative structural and functional characterization's usefulness in identifying potential research gaps is directly proportional to its depth and robustness, that is, to the degree to which it can detect variations in important quality characteristics between the biosimilar and the reference biologics (including additives and impurities), both qualitatively and quantitatively. For example, comparisons that reveal some or no difference between the biosimilar component and reference biologics will enhance the scientific case for ensuring biosimilarity. It can be advantageous to assess the similarities and assessments between the two components using a relevant retrospective analysis methodology that includes a large number of extra product parameters and the combination of them with high sensitivity utilizing orthogonal methodologies. This procedure may lessen the potential for unrecognized structural changes between products, resulting in a more specific and focused approach to clinical studies. A thorough knowledge of the biosimilar's mechanism of action (MOA) and the clinical significance of any witnessed structural differences, clinical knowledge of the reference biologic and its process indicating overall potential drawbacks, and the data relevant to pharmacodynamic (PD) measure(s) can assure additional evidence in significantly proving biosimilarity [1,2,9].

Current Strategy for Structural Analysis Demonstrating Biosimilarity

Sponsors should employ acceptable analytical procedures with enough acceptability to evaluate the protein structure. Usually, such results comprise the portfolio between the component being considered and the reference biologics: preliminary structures, such as amino acid sequence, long chain structures, including supplementary structures [6].

Sponsors must conduct a thorough structural analysis of both the component and reference biologics across several examples to comprehend the lot variation of all products throughout their manufacturing process. The lots utilized for analysis must demonstrate biosimilarity between the clinical specimens used in the research and the product of the referenced biologics. The analysis of batches produced in the establishment of procedures for the intended product might also be beneficial. Sponsors should explain their selection of relevant lots, specifying the total number of lots.

Advances in analytical techniques and technologies have revolutionized biosimilar characterization, enabling more precise and comprehensive evaluation of biosimilar candidates. High-resolution mass spectrometry (HRMS) and next-generation sequencing (NGS) enable detailed analysis of protein structure and genetic variations. Advanced chromatographic techniques, such as ultra-high performance liquid chromatography (UHPLC), enhance separation and detection of subtle differences in biosimilar molecules. Biophysical characterization tools, such as circular dichroism (CD) and nuclear magnetic resonance (NMR) spectroscopy, provide insights into higher-order structure and dynamics. Computational modeling and simulation approaches, such as molecular dynamics (MD) and machine learning algorithms, facilitate the prediction and analysis of biosimilar behavior.

These innovations enable more accurate and efficient comparison of biosimilars to reference products, ensuring equivalent safety, efficacy, and quality. By leveraging these cutting-edge technologies, the biosimilar industry can accelerate development, reduce costs, and ultimately enhance patient access to life-saving treatments [10].

Furthermore, EMA suggests that manufacturers examine the finished product characteristics of numerous components and reference products, evaluating additives and any product aspects on purity, product-focused and process-related contaminants, and stability. Formulation discrepancies between the component and comparative biologics might impact animal or clinical testing requirements [7,10].

Current Strategy for Functional Analysis Demonstrating Biosimilarity

The clinical efficacy of biosimilar products must be assessed via in vitro and/or in vivo evaluations. Biological and binding tests, as well as enzyme kinetics, are examples of in vitro experiments. In vivo tests may use animals of illness to assess impacts on pharmacodynamic indicators or effectiveness. Using functional assays to compare a component of interest to a referenced biologic is crucial for demonstrating biosimilarity and justifying selective animal and clinical testing [6,10].

Manufacturers can use these studies to ensure additional proof that the proposed product's activity and potency are similar to those of the reference biologic and to assure the idea that there are no clinically significant distinctions between the product and the referenced biologics. Such tests can also be used to give further proof that the MOA of both products is identical, to the extent that the MOA of the referenced product is available. Functional experiments may be used to supplement structural investigations by providing new data, investigating the ramifications of discovered structural variations, and exploring structure-activity correlations. These strategies may help evaluate analytical data and determine if more testing is needed to demonstrate biosimilarity [9,10].

Concept of interchangeability and its current standpoint in EU member states

Interchangeability is defined as the ability to exchange a drug for another that is anticipated to provide a similar therapeutic effect. EMA suggests that if a biosimilar is authorized by member states, it is interchangeable, which indicates that it can be utilized instead of its referenced product or the biosimilar can be substituted with another biosimilar of the same reference product. Interchangeability can only occur after a thorough examination of the permitted conditions of utilization, including checking the most current product characteristics [11].

Physician and patient perceptions play a crucial role in shaping interchangeability practices in the adoption of biosimilars. Physicians' confidence in biosimilars safety and efficacy influences their prescribing decisions, while patients' trust and understanding of biosimilars affect their acceptance and adherence. Physicians perceptions are shaped by factors such as familiarity with biosimilars, clinical experience with biosimilar-treated patients, access to accurate information and education, and concerns about potential immunogenicity or reduced efficacy.

Patients' perceptions are influenced by understanding their condition and treatment options, trust in their physician's recommendations, concerns about safety, efficacy, and potential side effects, and personal experiences with biosimilars or reference products.

Addressing these perceptions through education, open communication, and transparent information is essential to foster trust and confidence in biosimilars, ultimately shaping interchangeability practices and promoting optimal treatment outcomes. By understanding and addressing these factors, healthcare stakeholders can facilitate a smoother transition to biosimilars and enhance patient care.

The approach to biosimilar interchangeability differs greatly among EU member states. Some nations have implemented automatic replacement rules, while others need medical permission or patient approval before substitution. At present, Germany is a prime example of biosimilar substitution at the pharmacy level, with pharmacists regularly swapping biosimilars for reference pharmaceuticals unless expressly advised otherwise. In France, substitution is normally subject to physician approval, whereas in the United Kingdom, the approach to interchangeability is governed by municipal guidelines, with variations in practice [11,12].

Factors influencing patient access to biosimilars in the EU market

Patient access to biosimilars in the EU is influenced by various factors, including regulatory policies, healthcare system structures, and market dynamics. Biosimilars are often less expensive than reference biologics, increasing patient access by lowering out-of-pocket costs and overall healthcare expenses [13,14]. The availability of biosimilars, as well as their market share, can have an impact on patient access. As more biosimilars enter the market, competition frequently results in lower prices and greater availability [7,14]. Physicians have an important role in deciding patient access to biosimilars. Some member states may require physicians to convince them of the biosimilar's efficacy and safety before prescribing it, thereby restricting its availability [13,14]. Encouraging healthcare professionals to learn about biosimilars and their benefits can improve patient access. Training programs and information campaigns help providers understand when and how to successfully prescribe biosimilars [14]. Raising patient awareness of biosimilars can boost acceptance and access. Patients can learn about biosimilars' benefits and safety through informational campaigns and talks with healthcare practitioners. Some patients may be concerned about transitioning from reference biologics to biosimilars. Addressing these issues through education and communication can help improve access [9].

Patient access to biosimilars in the EU is gradually improving due to regulatory approvals, cost savings, and increased awareness. However, access varies greatly according to national policies and regional practices. Ongoing efforts to align policies, educate healthcare professionals, and address market difficulties are critical to ensuring widespread and fair access to biosimilars throughout the EU [15].

Market analysis

**Figure 2:**
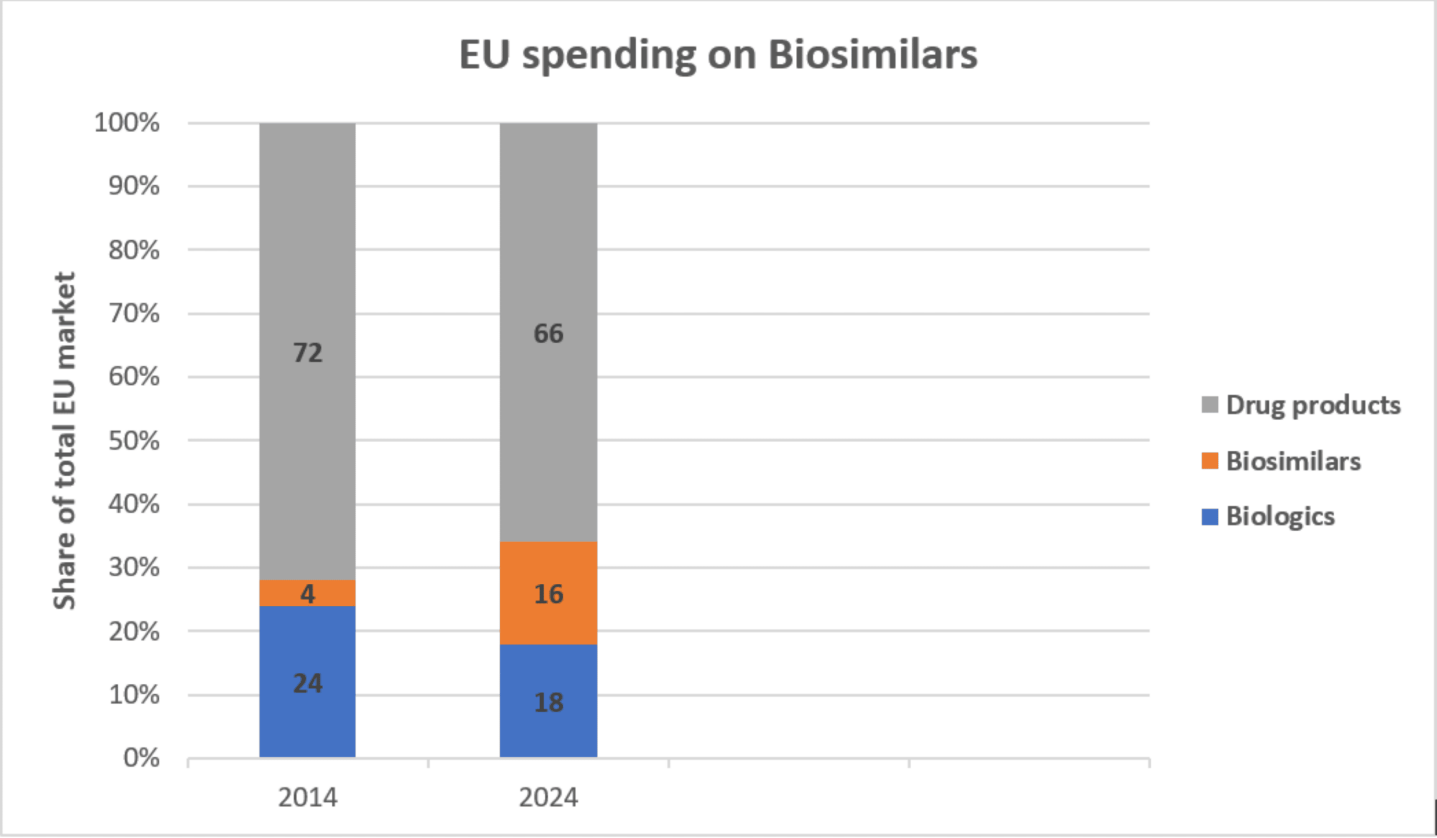
European market growth analysis This image was created by Mr. Gokul S.

Biosimilars are becoming an increasingly significant component of pharmaceutical spending due to their effectiveness as a therapy for complicated illnesses. Biosimilars account for 16% of total medication expenditure in Europe at list prices, and the biosimilars market within the EU has seen a higher growth rate compared to the broader biologics market. The compound annual growth rate (CAGR) for biosimilars in the EU has been approximately 15%-20% over the past five years. This accelerated growth is driven by increasing acceptance of biosimilars, cost-containment measures, and supportive policies [16], which is shown in Figure 2.

Successes of the biosimilar revolution

The biosimilar revolution in the EU has made significant advancements in delivering economical and effective treatment choices, but it still confronts major hurdles. Opportunities for further development can increase biosimilars' impact on healthcare systems. A summary of the achievements, problems, and potential for future development are as follows: Biosimilars have improved patient access to medicines for complicated disorders, including cancer, autoimmune diseases, and diabetes, by providing cost-effective alternatives to pricey reference biologics. Biosimilars have resulted in substantial savings for healthcare systems. These savings have enabled investments in other areas of healthcare, easing the financial burden on individuals and the public health system. Real-world data and post-market monitoring have shown that biosimilars are both safe and effective, supporting their position in clinical practice and increasing trust among healthcare professionals. Several EU nations have adopted biosimilar-friendly policies, including preferential reimbursement and substitute procedures. This has helped them integrate into healthcare systems.

Challenges of the biosimilar revolution

There is a lack of agreement among EU member states on biosimilar interchangeability and substitution. This diversity may lead to misunderstandings and impact the consistency of biosimilar usage. The standards for biosimilar replacement differ by country, which might impact biosimilar adoption and availability. An example includes regulations in Germany and France. Some healthcare practitioners are cautious about biosimilars because of worries regarding their effectiveness and safety when compared to reference biologics. This hesitation may have an impact on prescription habits. Patients may be hesitant about switching to biosimilars due to a lack of awareness or disinformation regarding their equivalence to reference products. Access to biosimilars varies by location, with some enduring delays or restricted availability owing to supply chain constraints or market factors. Ongoing patent challenges and reference biologic exclusivity terms might cause biosimilars to be delayed in entering the market and can lead to reduce the market share of biosimilars [1,7,14,17].

Opportunities for further development

The biosimilar revolution in the EU has had major benefits in terms of enhancing treatment access and lowering prices, but it still confronts obstacles in policy heterogeneity, education, and market access. Addressing these difficulties via harmonized regulations, greater education, and better market practices creates a potential for continued progress, eventually benefiting patients and healthcare systems throughout the EU.

Creating consistent rules and regulations throughout the EU may help to eliminate inefficiencies and speed up biosimilar approval and substitute processes. This may serve to decrease uncertainty and encourage widespread adoption. Implementing thorough biosimilar training programs for healthcare practitioners may help reduce skepticism and encourage informed prescription. Targeted education initiatives may increase patient knowledge and comprehension of biosimilars, improving acceptability and reducing switching concerns. Improving supply chain efficiency and dependability may help guarantee that biosimilars are consistently available across geographies. Promoting competition among biosimilars may reduce costs and make them more accessible to patients. Encouraging the development of novel biosimilars and broadening their therapeutic applications may increase treatment alternatives while lowering costs. Supporting research into biosimilars and their real-world effects may provide useful evidence to confirm their advantages and encourage greater use. Simplifying and clarifying regulatory procedures for biosimilars may speed up market entrance and lower hurdles for producers. Strengthening cooperation among regulators, manufacturers, and healthcare providers may help to enhance the regulatory process and integrate biosimilars into treatment recommendations.

Discussion

To address the challenges and opportunities in the biosimilar revolution, policymakers should develop clear, harmonized regulations for biosimilar approval and interchangeability and implement education and training programs for healthcare professionals and patients. They should also encourage price competition and transparent pricing models and establish robust pharmacovigilance systems to monitor biosimilar safety.

Manufacturers should invest in high-quality manufacturing processes and quality control measures and develop robust analytical and clinical data packages for biosimilar approval. They should engage in transparent communication with stakeholders about biosimilar development and approval and collaborate with healthcare providers and patient groups to address concerns and promote education.

Healthcare providers should stay up-to-date with the latest biosimilar research and clinical data and educate patients about biosimilar benefits, risks, and treatment options. They should develop clear treatment protocols and guidelines for biosimilar use, monitor patient outcomes, and report adverse events to pharmacovigilance systems. By working together, these stakeholders can ensure that biosimilars are developed, approved, and used in a way that benefits patients and the healthcare system as a whole.

The EU's biosimilar approach has far-reaching global implications, poised to shape the trajectory of biosimilar adoption in other regions. As a pioneer in biosimilar regulation, the EU's framework serves as a model for other countries to follow. The EU's emphasis on rigorous scientific evaluation, transparent decision-making, and post-market monitoring sets a high standard for biosimilar development and approval. This approach will likely influence regulatory agencies in other regions, such as the USA, Asia, and Latin America, to adopt similar stringent guidelines, ensuring consistent quality and safety standards globally. Moreover, the EU's success in promoting biosimilar competition and reducing healthcare costs may encourage other countries to adopt similar policies, driving increased adoption and access to biosimilars worldwide. As the global biosimilar landscape continues to evolve, the EU's approach will remain a key driver of progress, shaping the future of biosimilar development, approval, and adoption across regions.

## Conclusions

The EU has revolutionized the biopharmaceutical landscape, prioritizing patient needs and fostering innovation and competition. By embracing biosimilarity, interchangeability, and patient access, the EU has created a paradigm shift in the way we approach biosimilar treatments. The rigorous regulatory framework, built on biosimilarity and interchangeability guidelines, has established a gold standard for global biosimilar development. The market has undergone a significant transformation, with biosimilars capturing substantial market share due to growing acceptance and trust. Patent expiries and increasing competition have fueled price reductions and expanded treatment options, making biosimilar treatments more accessible to patients. Innovative pricing models and value-based agreements are redefining market dynamics, offering new opportunities for growth and evolution.

Despite these successes, challenges persist, including education and awareness gaps among stakeholders, concerns regarding the extrapolation of indications, and the need for real-world evidence to support long-term safety and effectiveness. However, opportunities for growth and evolution abound, including the integration of real-world data and digital health technologies, refining interchangeability guidelines, and exploring innovative pricing models and value-based agreements. Ultimately, the EU's biosimilar resolution serves as a model for global adoption, demonstrating the potential for biosimilars to enhance patient access and affordability, drive innovation and competition, and optimize healthcare resource allocation. As the biosimilar landscape continues to evolve, the EU's approach will remain a guiding force, shaping the future of biologic treatments and improving patient lives.
